# Life at the mesoscale: the self-organised cytoplasm and nucleoplasm

**DOI:** 10.1186/s13628-015-0018-6

**Published:** 2015-02-25

**Authors:** Richard P Sear, Ignacio Pagonabarraga, Andrew Flaus

**Affiliations:** Department of Physics, University of Surrey, GU2 7XH Guildford, Surrey UK; Departament de Fisica Fonamental, University of Barcelona, 08028 Barcelona, Spain; Centre for Chromosome Biology, School of Life Sciences, National University of Ireland Galway, Galway, Ireland

**Keywords:** Workshop report, Self-organisation, Mesoscale, Cell biology, Cytoskeleton, Cytoplasmic bodies, Chromatin

## Abstract

The cell contains highly dynamic structures exploiting physical principles of self-organisation at the mesoscale (100 nm to 10 μm). Examples include non-membrane bound cytoplasmic bodies, cytoskeleton-based motor networks and multi-scale chromatin organisation. The challenges of mesoscale self-organisation were discussed at a CECAM workshop in July 2014. Biologists need approaches to observe highly dynamic, low affinity, low specificity associations and to perturb single structures, while biological physicists and biomathematicians need to work closely with biologists to build and validate quantitative models. A table of terminology is included to facilitate multidisciplinary efforts to reveal the richness and diversity of mesoscale cell biology.

## Background

The workshop “Life at the mesoscale: The self-organised cytoplasm and nucleoplasm” took place over 3 days in July 2014 at the home of CECAM (Centre Européen de Calcule Atomique et Moléculaire) in Lausanne, Switzerland. It brought together 30 physicists, molecular cell biologists and mathematicians with the aim of combining skills and approaches to study self-organisation in the cytoplasm and nucleoplasm of cells. The workshop was organised by Richard Sear, Trevor Dale, Andrew Flaus and Ignacio Pagonabarraga.

Traditionally, the cytoplasm has been viewed as a well mixed and spatially homogeneous mixture of monomers and small complexes. Similarly, the nucleoplasm has been viewed as a uniform chromosome “spaghetti”. However, rapid developments in light microscopy and “omics” data correlations are giving us unprecedented access to cell structure and dynamics on mesoscale length scales.

**Figure 1:**
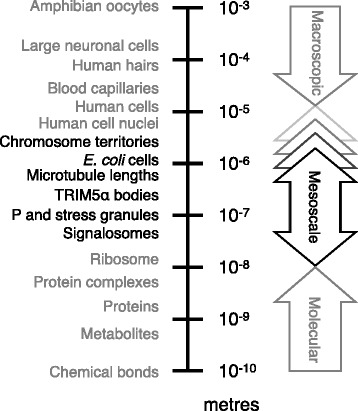
**Mesoscale lengths in context.** The self-organised structures considered in the workshop are mesoscale structures. We define mesoscales as length-scales larger than discrete molecular complexes yet remaining intracellular. These limits are bounded below by ribosomal diameters (25 nm) as the molecular scale, and above by typical model cell diameters. For *E. coli*, this is approximately 1 μm, while for human cells it is of order 10 μm. Unusually large cell diameters such as neurons (100 μm) and amphibian oocytes (1 mm) mean the upper limit will depend on the cell.

**Figure 2:**
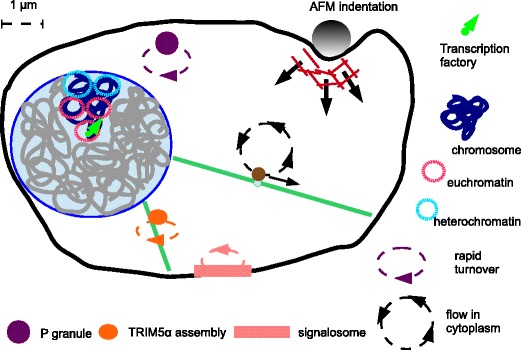
**Schematic of a eukaryote cell illustrating self-organised structures in both the cytoplasm and the nucleoplasm.** In the nucleus we highlight (in dark blue) a single chromosome, restricted to its territory, and show this chromosome’s euchromatin and heterochromatin domains. In the cytoplasm we have shown a number of cytoplasmic bodies: P granules, TRIM5α assemblies and a signalosome. In all three cases we have used a dashed ellipse with an arrow to indicate that at least some components of the body turn over rapidly, in minutes or less. Finally, we have also indicated microtubules (green) and actin filaments (red). We have shown flow of the cytoplasm due to a bulky cargo (brown) being pulled along a microtubule, and the cell’s actin-based cortex deforming as a bead is pushed down onto the cell.

Here we define the mesoscale as being length scales larger than individual molecular machines such as ribosomes, but no larger than the size of the cell. In Figure 1 we place this range of length scales in context. With new experimental techniques we can see that on these length scales the cytoplasm and nucleoplasm are neither uniform nor static, but are highly organised and often highly dynamic. We have been dramatically underestimating the extent of this mesoscale self-organisation, and its role in key processes of the cell function. As our understanding increases, the number of mesoscale structures we are aware of is growing. We have illustrated a selection of the structures discussed at the workshop in Figure 2. This figure illustrates “mesoscale cell biology” in the sense that it is organisation of the cell interior on mid-range length scales.

The structures illustrated in Figure 2 are self-organised. Their formation is due to their molecular components physically interacting and consuming energy, not due to external forces. Proteins, RNA, and DNA molecules self-organise into a wide variety of clusters, bodies, cages, domains, territories and long range networks that are often highly dynamic; proteins may have residence times in these assemblies of less than a minute. Many of these assembles can be dynamically formed and remodeled depending on signals and cellular conditions, and these processes typically involve active processes that consume energy.

To properly understand this mesoscale self-organisation requires collecting high resolution observations and combining them with quantitative, predictive models. We believe that collaboration among cell biologists, biological physicists and mathematicians will be essential to achieve this. The workshop aimed to catalyse this collaboration.

CECAM is a centre for organising workshops based at EPFL (Ecole Polytechnique Fédérale de Lausanne), in Lausanne, Switzerland. The centre has its origin in the field of the computer simulations of liquids but now organises workshops on a variety of topics that include a significant component of computational physics or chemistry. In this meeting report, we will start by highlighting the problem of scientists from different backgrounds using different terminology for highly analogous structures, or the same word for structures with rather different properties. We will then summarise the key points discussed in the workshop. We end with a short summary of what the workshop concluded were the most important advances required in this new field of mesoscale biology.

## Discussion

### Language at the interface between biology and physics

The workshop location in multilingual Switzerland was particularly apt given the tendency of physicists and biologists to speak different languages. The speakers worked to overcome these language barriers, and there were open discussions where biologists focussed on general principles in life’s myriad variations, while physicists clearly spelled out the meaning and implications represented by formal approaches.

This need for a mutually understandable shared language was highlighted by discussions on how physicists and biologists may use the same word to mean different things. To help avoid this problem, in Table 1 we have provided a short list of some useful terms. For example, a word that received attention was the simple label “aggregate”: Some biologists tend to use “aggregate” to describe non-functional associations, including possible artefacts of overexpression, and dangerous destinations for misfolded biomolecules. Physicists tend to use the term as a convenient holding place for associations of indeterminate structure. We therefore recommend that the word “assembly” be used in preference to “aggregate” for bodies or clusters of many proteins and other biomolecules in cells that are assumed or known to be related to function.

**Table 1:** **Primer of terminology relevant to mesoscale cell biology**

**Active matter**	Matter that is consuming energy, such that its dynamics or structure changes significantly if the energy supply is removed.
**Aggregate**	Many molecules adhering together into a body or cluster, often in an undesirable, non-functional, or poorly characterised state. This can involve strong interactions resulting in a body that is not in dynamic equilibrium with the surroundings, so molecules are not able to continuously leave and be replaced. Aggregate can also refer to the process of formation of the body of molecules.
**Assembly**	Bodies or clusters of many proteins and other biomolecules in cells that are assumed or known to be related to function. Assembly can also refer to the process of formation of these bodies or clusters, as in self-assembly (see below).
**Ballistic motion**	Motion in a straight line at constant speed. The term ballistic motion can be misleading for dynamics inside a cell because inertia is irrelevant at these sizes, and a cellular component only moves at a constant speed if a force is continuously applied that exactly matches the drag experienced by it.
**Cell cortex**	Organisation of inner plasma membrane and underlying cytoskeleton creating a layer capable of contraction. The cortex controls cell morphology and facilitates movement.
**Chromosome territory**	A volume of the nucleus inhabited by a single chromosome, with relatively little overlap with the volumes occupied by other chromosomes. This contrasts with an intermingled chaotic mixture.
**Colloid**	Molecules or polymolecular particles dispersed in a liquid, where the particles have a dimension of 1 nm to 1 μm in at least one direction.
**Cytoplasmic body**	A liquid-like droplet in the cytoplasm that is strongly enriched in a set of biomolecules, at least some of which are in dynamic equilibrium with the cytoplasm. Cytoplasmic bodies may deform and flow as viscoelastic liquids under force, but unlike liquids their the size and number are limited. These bodies are referred to by a variety of terms including assemblies, granules, clusters and aggregates, or named for their function. They are not membrane bound.
**Cytoskeleton**	Scaffold of molecular filaments in eukaryotic cells including microfilaments (actin), microtubules (tubulin) and intermediate filaments (keratin, lamin). This scaffold is not static and is constantly being dynamically remodeled.
**Euchromatin and heterochromatin**	Linear regions of open (euchromatin) or compacted (heterochromatin) nucleosomes along interphase chromosomes. They may come together in the nucleus and be identifiable by light microscopy via characteristic histone modifications or accessory proteins. Histones are proteins that bind DNA in eukaryotes to form structures called nucleosomes that are around 10 nm in diameter.
**HiC methodology**	A type of chromosome conformational capture (3C) that enables unbiased sampling of the close association of any site in the genome with every other site. It uses chemical crosslinking of DNAs, enzymatic cleavage and re-ligation of DNA, followed by massively parallel sequencing of ligated DNA pairs.
**Mean-field theory**	A class of theory where fluctuations around a mean value are ignored. For example, the concentration of a cell signaling molecule may be fluctuating around a steady state value, but a mean field theory neglects these fluctuations and uses a constant steady-state estimate.
**Mesoscale**	A length scale larger than molecular scales of 1 to 10 nm but no larger than the size of the cell. In a cell, mesoscales are approximately 100 nm to the typical 10 μm cell diameter.
**Multiscale systems**	Systems with important structure and dynamics at multiple length and time scales. For example, the mitotic spindle has dynamics for tubulin joining a growing microtubule as well as for whole chromosomes.
**Non-equilibrium phenomena**	Phenomena characteristic of systems where any of the following are true: The system is consuming energy; external forces are acting on the system; the system has not yet reached its final equilibrium state.
**ODE, PDE**	An ordinary differential equation (ODE) is an equation with differentiation in only one variable, often time. A partial differential equation (PDE) has 2 or more variables, often time and one or more of the 3 spatial dimensions x, y and z.
**Phase diagram**	Typically a plot in which axes represent control parameters (e.g. temperature, concentration), and where in the plot there are areas of two or more distinctive behaviours such as molecules clustering into bodies or spread uniformly. A transition line separates these areas, and denotes the conditions where the system switches between the behaviours.
**Phase separation**	Separation of any fluid spontaneously into two or more distinct fluids without an energy consuming process, e.g. as oil and water separate. Neither phase is pure so some molecules remain dissolved in the complementary phase. Although phase separation may start with small droplets of one phase in the other, these droplets grow until one liquid layers on top of the other at equilibrium.
**Self-assembly**	The process of spontaneous formation of structures driven by intermolecular interactions without a requirement for energy consumption. For example, surfactant micelles and clathrin lattices.
**Self-organisation**	Formation of structure or coordination that occurs spontaneously in a system such as a cell. The organisation arises due to processes inside the cell and is not externally imposed. In cells it arises due to intermolecular interactions or energy-consuming processes. Self-organisation is more general and larger in scope than self-assembly.
**Soft-matter physics**	The physics of matter that is mostly either liquids, or soft solids that can be made to stretch or flow under relatively small forces. Soft matter also conventionally includes polymers, even though these may not always readily flow.
**Transcription factory**	Putative multi-protein enzyme structure including RNA polymerases. These transcribe multiple RNA molecules such as messenger RNA precursors, from multiple genes, and these genes may move to the factory to be transcribed. This movement of genes to factories contrasts with textbook models of RNA polymerase moving to assemble and translocate along a static DNA template.
**Viscoelasticity**	Viscoelastic liquids such as the cytoplasm and nucleoplasm are liquids intermediate in behaviour between simple liquids and solids. Unlike solids they do flow when forces are applied, but unlike simple liquids the speed at which they flow is not simply proportional to the force exerted. Applying the same force rapidly or slowly may have different effects.

The enthusiastic interactions in the workshop illustrate the importance of dialogue in this multidisciplinary field, and the value of open discussions to tease out confusion and ensure that insights from multiple perspectives can be combined.

### Liquid-like assemblies in the cytoplasm

One focus of the workshop was on functional assemblies that, at least superficially, resemble liquid droplets in the cytoplasm. This began with Georg Stoecklin (German Cancer Research Centre, Heidelberg), who described the phenomena of P bodies and stress granules [1]. These related assemblies act to concentrate components of the RNA processing machinery, Also, in the case of stress granules, they form rapidly in response to stress. The bodies illustrate key generic properties of mesoscale structures. These include relatively uniform, and so presumably controlled, size and number; dynamic formation; an ability to fuse; and rapid turnover of component factors. This checklist of characteristics may help us define a class of self-organised assemblies in cells, and suggest standard ways of modelling them.

Chiu Fan Lee (Imperial College London) complemented this example with a description of P granules in *C. elegans* germ line cells [2]. He pointed out that these undergo asymmetric localisation during cell division under the control of a gradient of the protein MEX-5 in the cell. Lee and colleagues have modelled this as a phase separation phenomenon [3].

Melissa Gammons (Medical Research Council Laboratory of Molecular Biology, Cambridge) looked at the role of assemblies in cell signaling, specifically the Wnt pathway. She discussed “signalosomes” formed of a number of signaling proteins including the reversibly polymerising protein Dishevelled [4]. Gammons also started discussion of using super-resolution techniques such as STORM to probe these assemblies at high resolution. This resolution is needed to see if bodies are indeed relatively uniform and liquid-like, or have substantial structure on length scales smaller than the wavelength of light.

In contrast to bodies with multiple components, Ed Campbell (Loyola University, Chicago) described the family of TRIM proteins that form possibly simpler assemblies. He focused on the TRIM5α protein whose function is to restrict HIV. TRIM5α forms bodies that appear to encapsulate virions for disassembly [5]. This work illustrates the importance of coupling cell biological observations with detailed molecular structure-function analyses to understand the molecular mechanism of self-assembly of mesoscale bodies.

Mesoscale self-association patterns are not always functional, which was illustrated by Giuseppe Foffi’s talk (Université de Paris-Sud, Orsay) on the highly concentrated solution of the crystallin proteins inside the cells of the lenses of our eyes [6]. Cataracts result from the formation of liquid droplets in these cells. The concepts of solution phase behaviour were also presented by Ilja Voets (Eindhoven University of Technology), who described observations of mixtures of proteins. In these mixtures, in some cases different protein species can attract each other and so separate out together from a dilute solution, or in other cases they repel each other and separate into coexisting solutions, with each solution rich in one protein [7]. This work on the formation of liquid droplets in simple *in vitro* protein solutions can help us understand the liquid-like structures that form in the much more complex, and energy consuming, mixtures that are inside cells.

### Self-organisation of the cytoskeleton as an active material

Perhaps the best studied and most well known examples of mesoscale structures in cells come from the cytoskeleton. A number of speakers addressed aspects of cytoskeletal self-organisation. Guillaume Charras (University College London) introduced a “poroelastic” model which describes the cytoplasm, for short times and high frequencies, as an elastic mesh in a viscous fluid [8]. This mesh comes at least in part from the cytoskeleton.

Isabel Palacios (University of Cambridge) took the cellular significance of the cytoskeleton a step further in describing the active phenomenon of cytoplasmic streaming. This fluid flow acts as a “molecular stirrer” in *Drosophila* oocytes. The flow relies not only on microtubules and the molecular motor kinesin, but is sensitive to the actin cytoskeleton mesh [9].

It is well known that kinesin and myosin motors can actively drive mesoscale bodies into non-equilibrium arrangements, and several speakers outlined efforts to understand the properties and model the behaviour of these motors. Stefan Diez (Dresden University of Technology) described detailed biochemical understanding of kinesin motions. He discussed not only their well known contribution to cargo transport, but also new thinking about potential contributions to the elasticity of the microtubule network [10]. This discussion of the role of molecular motors was subsequently complemented by Rhoda Hawkins (University of Sheffield) who presented a mathematical model for motors generating elastic behaviour as an “active solid” [11].

Another direction in thinking about the active properties of the cytoskeleton was presented by Gijsje Koenderink (Foundation for Fundamental Research on Matter Institute for Atomic and Molecular Physics, Amsterdam), who presented *in vitro* work on myosin-mediated self-organisation of actin networks [12]. Later in the workshop, Andrea Parmeggiani (University of Montpellier) explained how simple traffic-on-network models can be used to model cytoskeletal motor networks [13]. An elegant example of modelling an important cellular process was provided by Jean François Joanny (Ecole Superieure de Physique et de Chimie Industrielles de la Ville de Paris, ParisTech). He considered the actin contractile ring in cytokinesis [14], and showed how the force generation and dynamics of the cytoskeleton can be quantitatively modelled.

### Self-association and order in the nucleoplasm

Although the nucleus often appears relatively homogenous in low resolution microscopy images, the more quantitative data we acquire, the more self-organisation we see. This new flow of data is exemplified by genome-wide technologies such as HiC that provide very detailed quantitative data on the structure of chromosomes. Such detailed information is not yet available for structures in the cytoplasm. This approach was illustrated by Peter Fraser (Babraham Institute, Cambridge) who described how single cell genome structure studies reveal organisational patterns within decondensed chromosomes. Chromosomes are clearly highly structured. For example, transcription is not uniform in time and occurs in bursts for most genes. It is also not uniform in space, for example when organised into transcription factories [15]. It is clear that functional self-organisation at the mesoscale is as important in the nucleoplasm as it is in the cytoplasm.

Related to this, Karen Lipkow (Babraham Institute, Cambridge), outlined methods and correlations of expression pattern and protein binding characteristics. Histone proteins provide the foundation nucleosome unit underlying eukaryotic chromatin structure, so the histone diversity and dynamics described by Andrew Flaus (National University of Ireland Galway) allows us the opportunity to understand the molecular basis of eukaryotic chromatin organisation from the ground up. Nucleosomes are not homogenous and static units, but display local patterns of chemical features whose distribution can be dynamically altered by enzymes that modify, remodel or replace them. This perspective opens the potential to interpret chromatin as a dynamic polymeric network with tuneable local variations on the molecular scale that can propagate to higher levels of assembly. Such an interpretation can incorporate genome-wide observations of variations at a regional scale, and contribute to understanding the mechanism for action at a distance in genomic processes.

In contrast with the early status of models for eukaryotic chromatin encased in the membrane-bound nucleus, the nucleoid region of *Escherischia coli* has already received detailed attention. Marco Cosentino Lagomarsino (University Pierre and Marie Curie, Paris) described the viscoelastic properties and complex dynamics exhibited by the nucleoid [16].

### The immediate challenges

The workshop ended with reflections by Helen Byrne (University of Oxford). She used Wnt signalling to illustrate the challenges of modelling biological processes [17]. She noted that it can be straightforward to develop complex models, but then the challenge shifts to obtaining the experimental data required to adequately constrain the parameters of the model. This reflection nicely illustrates the importance of good communication of aims, needs and findings between those doing experiments and those developing models.

## Conclusions

We would like to end this report as we did the workshop, by considering what developments would significantly advance the field of self-organisation in the cytoplasm and the nucleoplasm. The participants came up with five:

1. Imaging improvements such as new super-resolution microscopy technologies to observe individual actin filaments and microtubules in live cells, in order to understand the dynamics at the single filament level.
2. Genomics and microscopy data for chromosome dynamics on 10 nm to 1 μm length scales, and at the associated time scales, in order to model genome processes.
3. Quantitative biochemical cataloguing and modelling of the internal structure and composition of cytoplasmic bodies within cells.
4. Development of approaches that allow targeted perturbations to probe spatiotemporal dynamics within cells, such as optogenetics
5. Expansion of opportunities for like-minded biologists and physicists with interests in related areas to meet and develop shared understanding and initiate collaborations.

These advances are essentially the acquistion of better experimental datasets, and the improved use of this data to build and validate quantitative models of self-association and motor-driven function at the mesoscale. The underlying challenge is to observe and understand the highly dynamic, low affinity and low specificity associations of the self-organised cytoplasm and nucleoplasm. Fulfilling this goal will naturally require close multidisciplinary interactions between biologists and physicists to understand the forces and processes involved, and see both embracing the fascinating richness and diversity of life at the mesoscale.

## Abbreviations

TRIM: Tripartite motif family
CECAM: Centre Européen de Calcule Atomique et Moléculaire
EPFL: Ecole Polytechnique Fédérale de Lausanne
MEX-5: Muscle excess 5 protein
STORM: Stochastic optical reconstruction microscopy
HIV: Human immunodeficiency virus
3C: Chromosome conformational capture
HiC: High resolution chromosome conformational capture
ODE: Ordinary differential equation
PDE: Partial differential equation
